# Note from the Editor: Looking Forward

**Published:** 2006-01

**Authors:** James G. Burkhart

**Affiliations:** Acting Editor-in-Chief, *EHP,* National Institute of Environmental Health Sciences, National Institutes of Health, Department of Health and Human Services, E-mail: burkhart@niehs.nih.gov

With this issue *EHP* bids a warm goodbye to Tom Goehl, our Editor-in-Chief since October 2001. Tom is a true altruist, always believing that the role of *EHP* is to impact the human condition by providing a forum for scientific information to be used by researchers, policy makers, and individuals to improve human health around the world. His devotion, drive, and integrity in working toward this goal are unmatched. I have no doubt that Tom will continue to be a dedicated contributor to global environmental health, though he claims it may be from a secluded camping spot far, far away. Farewell and safe journeys!

The January 2006 cover introduces a new look for the face of *EHP*. Over the last few years we have received many compliments on the cover photography and graphic art appearing with each new issue. These images have always been designed to convey a visual link to an environmental health topic in each issue. The new covers will continue in this tradition with some modifications we hope will enhance usability, mainly by providing in the left margin a way for the reader to quickly scan major news and research topics in the issue. The cover titles will also be more descriptive. The Table of Contents will now appear solely inside the journal, leaving the back cover available for announcements or advertisements. As always, we welcome any comments and suggestions you may have.

The staff of *EHP* wishes each of you the very best in the coming year and success in meeting the challenges that will inevitably come our way. As a member of a dynamic global environmental health community, *EHP* will continually strive to provide you with the best information for improving human and global health.

